# Enhancement of Room-Temperature Low-Field Magnetoresistance in Nanostructured Lanthanum Manganite Films for Magnetic Sensor Applications

**DOI:** 10.3390/s22114004

**Published:** 2022-05-25

**Authors:** Nerija Zurauskiene, Voitech Stankevic, Skirmantas Kersulis, Milita Vagner, Valentina Plausinaitiene, Jorunas Dobilas, Remigijus Vasiliauskas, Martynas Skapas, Mykola Koliada, Jaroslaw Pietosa, Andrzej Wisniewski

**Affiliations:** 1 Center for Physical Sciences and Technology, 10257 Vilnius, Lithuania; voitech.stankevic@ftmc.lt (V.S.); skirmantas.kersulis@ftmc.lt (S.K.); milita.vagner@ftmc.lt (M.V.); valentina.plausinaitiene@chf.vu.lt (V.P.); jorunas.dobilas@ftmc.lt (J.D.); remigijus.vasiliauskas@gmail.com (R.V.); martynas.skapas@ftmc.lt (M.S.); mykola.koliada@ftmc.lt (M.K.); 2 Faculty of Electronics, Vilnius Gediminas Technical University, 03227 Vilnius, Lithuania; 3 Faculty of Chemistry and Geosciences, Vilnius University, 03225 Vilnius, Lithuania; 4 Institute of Physics of the Polish Academy of Sciences, 02-668 Warsaw, Poland; pietosa@ifpan.edu.pl (J.P.); wisni@ifpan.edu.pl (A.W.)

**Keywords:** colossal magnetoresistance, low-field magnetoresistance, manganite films, nanostructured thin films, MOCVD technology, magnetic field sensors

## Abstract

The results of colossal magnetoresistance (CMR) properties of La_1-x_Sr_x_Mn_y_O_3_ (LSMO) films grown by the pulsed injection MOCVD technique onto an Al_2_O_3_ substrate are presented. The grown films with different Sr (0.05 ≤ *x* ≤ 0.3) and Mn excess (y > 1) concentrations were nanostructured with vertically aligned column-shaped crystallites spread perpendicular to the film plane. It was found that microstructure, resistivity, and magnetoresistive properties of the films strongly depend on the strontium and manganese concentration. All films (including low Sr content) exhibit a metal–insulator transition typical for manganites at a certain temperature, *T*_m_. The *T*_m_ vs. Sr content dependence for films with a constant Mn amount has maxima that shift to lower Sr values with the increase in Mn excess in the films. Moreover, the higher the Mn excess concentration in the films, the higher the *T*_m_ value obtained. The highest *T*_m_ values (270 K) were observed for nanostructured LSMO films with *x* = 0.17–0.18 and *y* = 1.15, while the highest low-field magnetoresistance (0.8% at 50 mT) at room temperature (290 K) was achieved for *x* = 0.3 and *y* = 1.15. The obtained low-field *MR* values were relatively high in comparison to those published in the literature results for lanthanum manganite films prepared without additional insulating oxide phases. It can be caused by high *Curie* temperature (383 K), high saturation magnetization at room temperature (870 emu/cm^3^), and relatively thin grain boundaries. The obtained results allow to fabricate CMR sensors for low magnetic field measurement at room temperature.

## 1. Introduction

The increasing demand in magnetic field sensors and high-performance magnetometers has resulted in the rapid advance of various sensor technologies [1]. Different areas of sensor applications require diverse properties, from high sensitivity and detectivity for biomedical applications to a wide range of magnetic field sensing for industrial and scientific applications. Recent technological progress allows the fabrication of compact, small physical dimension sensors with increased sensitivity and reduced cost for mass production. It was predicted that the growth of the magnetoresistive (MR) sensor market will come at the expense of Hall Effect technology or combination with it due to higher sensitivity and more widespread applications [1,2]. Magnetoresistive sensors are also important for sensing oscillator methods, which have high sensitivity and accuracy [3,4,5]. Among commercially available MR sensors, the performance of which is based on such physical phenomena as anisotropic, tunneling, and giant magnetoresistance (AMR, TMR, and GMR, respectively) [1,6], the colossal magnetoresistance effect (CMR) is also promising for future sensor development technologies [7], including high-field applications [8,9]. For a long time, the main disadvantages of the CMR effect—high sensitivity to ambient temperature variations and low room-temperature sensitivity to the magnetic field—were the main obstacles in the development of CMR sensor technologies up to higher technological readiness levels (TRL) [10].

The most intensive studies were performed on doped perovskite manganites exhibiting the CMR effect [11,12]. It was demonstrated that polycrystalline manganites exhibit high magnetoresistance values in a wide range of temperatures lower than the phase transition from paramagnetic to ferromagnetic state, in contrary to good quality monocrystalline or epitaxial manganites, showing high MR values only in the vicinity of the *Curie* temperature [13]. Due to close interplay between structural, magnetic, and transport properties, the manganites have a number of features useful for magnetic sensor development, which can be tuned over wide range of temperatures and magnetic fields. It was demonstrated that nanostructured La_1−x_Sr_x_MnO_3_ (La-Sr-Mn-O, LSMO) manganite films can be used for the development of magnetic sensors, which are capable of measuring the magnitude of pulsed magnetic fields in very small volumes (so-called CMR-B scalar sensors) up to very high magnetic fields [8,14]. Such sensors were used for the magnetic field measurement during electromagnetic acceleration in railguns and superconducting systems [15,16], the magnetic field distribution in non-destructive pulsed-field magnets [17], and evaluation of welding quality during magnetic welding of metals [18]. The possibility to tune temperature and magnetic field ranges of operation of CMR sensors by changing deposition temperature and film thickness was demonstrated [19]. However, for conventional biomedical or industrial applications, sensors operating at room temperature and measuring much lower magnetic fields are required.

It was found that polycrystalline films exhibit a so-called low-field magnetoresistance (*LFMR*) effect, which is usually explained by spin-polarized tunneling of charge carriers in high structural quality crystallites through disordered grain boundaries (GBs) [20,21]. The *LFMR* exhibits the highest values at low temperatures and could be recognized by the abrupt change in film resistance under an applied weak magnetic field. Theoretically, it is predicted not to exceed ~33% [20,21]. However, for doped lanthanum manganites, the *LFMR* usually vanishes at room temperature. It was found that *LFMR* in manganites could be increased by special engineering of various boundaries in the films, including grain boundaries (GBs), phase boundaries (PBs), ferromagnetic domain boundaries (DBs), and interfacial effects between film and a substrate [22,23,24]. Usually, high *LFMR* values are associated with a large number of grain boundaries having noncollinear spin structure, good connectivity between the crystallites, and a high saturation magnetic moment of an individual crystallite [25]. The decrease in crystallite dimensions results in a higher amount of grain boundary material, which is desirable; however, it could cause the decrease in the saturation magnetic moment of the crystallites. A lot of efforts were undertaken to increase the *LFMR* by introducing the second insulating phase during the growth of the films or fabrication of thin film junctions with an insulating layer. For example, very high *LFMR* values up to 83% were obtained below 20 mT at 4.2 K for specially grown La_0.67_Sr_0.33_MnO_3_/SrTiO_3_/La_0.67_Sr_0.33_MnO_3_ thin film junctions [26]. As *LFMR* is largely controlled by grain boundary and interfacial effects, especially high *LFMR* values could be achieved in two-phase vertically aligned nanocomposite (VAN) films with well-ordered vertical phase boundaries [27]. Such VAN systems, usually composed of a ferromagnetic phase, such as La-Sr-Mn-O, and an insulating one, such as NiO, ZnO, CeO [28,29,30], etc., provide the possibility to tune their magnetoresistance values and other main parameters in a wide range depending on the relative amount of these phases. In [31], the authors give a comparison of *LFMR* values obtained from the literature in various nanocomposites and nanostructures as well as present their own results on synthesized nanoparticles exhibiting enhanced *LFMR* in comparison with large crystallites and bulk material. However, it has to be mentioned that the fabrication of nanocomposites with reliable properties requires advanced technologies, which could be difficult to adapt for commercial production. In [7], the authors demonstrated that the metal-organic chemical vapor deposition (MOCVD) is one of the preferred techniques for production of high-quality manganite nanostructures in a scalable and economic way. It allows to grow the self-formed vertically aligned one-phase manganite nanostructures with tunable parameters [19]. For room temperature applications, the films with increased phase transition temperature (*Curie*) are required. It is known that for good quality epitaxial La_1-x_Sr_x_MnO_3_ films, the “optimal” composition *x* ≈ 1/3 gives the highest *Curie* temperature (*T*_C_) values [11]. However, for polycrystalline or nanostructured films, *T*_C_ as well as metal–insulator transition temperature *T*_m_ could be affected by various other factors such as nonstoichiometric chemical composition, strain, film thickness, disordered grain boundary material, etc. 

In this paper, we present a comprehensive study of low-field magnetoresistive properties of single-phase nanostructured lanthanum manganite La_1−x_Sr_x_Mn_y_O_3_ films grown by the pulsed injection (PI) MOCVD technique with different Sr content (*x*) and Mn excess (*y*) and demonstrate the possibility to use these films for room temperature magnetic field sensor applications.

## 2. Experimental Details

### 2.1. Film and Sample Preparation

Lanthanum manganite La_1−x_Sr_x_Mn_y_O_3_ (LSMO) films with different Sr (*x*) content and excess of Mn (*y*) were deposited onto a polycrystalline Al_2_O_3_ substrate by using a PI MOCVD technique. The temperature of the substrate was kept constant (750 °C) during the growth process. The thickness of the films was estimated by using a profilometer and it was found in the range of (350–360) nm. As precursors, toluene-dissolved La(thd)3, Sr(thd)2, and Mn(thd)3 (thd is 2,2,6,6-tetramethyl-3,5-heptandionate) were prepared. A 2 Hz frequency was used to inject microdoses of approximately 3 mg of an organic solution with a mixture of dissolved precursors. The flash evaporation of the microdoses was performed at ~270 °C and the obtained vapor mixture was transported in the reaction chamber towards the heated substrate by a gas flow (~95 l/h) of argon and oxygen with ratio of 5:1, respectively. The growth rate of all investigated films was approximately 28 nm/min. Three groups of the films with different Mn excess *y* = Mn/(La + Sr) were prepared: the composition of precursors’ solution was chosen to obtain the films with constant content of Mn (*y* = 1.05, 1.10, and 1.15) while changing the composition of Sr (*x* = 0.05–0.3). To obtain a pure perovskite phase of the films, the deposition conditions were at first optimized by growing the films on monocrystalline LaAlO_3_ (LAO) substrates, and only after that the films were deposited on polycrystalline Al_2_O_3_ substrates. Moreover, both substrates (Al_2_O_3_ and LAO) were used during final growth of LSMO films with various compositions for further comparison of their properties. After the deposition of the films, the annealing at the same temperature (750 °C) for 10 min in a pure oxygen atmosphere was performed and then the films were slowly (with ~4.7 °C/min rate) cooled down to 350 °C. The samples were prepared by planar photolithography. For electrode formation, a Cr sublayer with Ag contact pads were thermally deposited. After that, the post-annealing was performed at 450 °C for 1 h in Ar atmosphere. 

### 2.2. Characterization

The characterization of the grown films was performed by the following techniques. The Inductively Coupled Plasma High-Resolution Mass Spectrometry (ICP-MS) was used to determine the composition of the films. To study the surface morphology of the films a Scanning Electron Microscope (SEM) (Hitachi SU70) was used. The crystalline structure of the films was investigated by using a Transmission Electron Microscope (TEM) (Tecnai G2 F20 X-TWIN) with an Energy Dispersive X-ray spectrometer (EDAX).

The resistivity *ρ* dependences on temperature were investigated by using a closed-cycle helium gas cryocooler (Janis 4K) in the temperature range of *T* = (5–300) K. The measurement of film’s magnetoresistance was performed in the same cryocooler positioning the sample holder between the poles of an electromagnet. The resistance vs. magnetic flux density (*B*) dependencies were measured in the temperature range 250–300 K up to 0.8 T. During the measurement, the temperature was kept constant with an accuracy of 0.1 K. The magnetoresistance was calculated according to the equation *MR(*%) = 100 × [*R*(*B*) − *R*(0)]/*R*(0), where *R*(*B*) and *R*(0) are field and zero-field resistance, respectively. Several samples prepared from the same film were measured for the statistical analysis.

Magnetic characteristic measurements were carried out in magnetic fields up to 50 kOe with an MPMS-5 SQUID (Quantum Design) magnetometer, and the dependences of the magnetic moment on magnetic field at selected temperatures were recorded. The magnetic hysteresis loops were measured after field-cooling at 1000 Oe in the temperature range from 320 K down to 220 K. Magnetization measurements were performed at the Institute of Physics of the Polish Academy of Sciences.

## 3. Results and Discussion

### 3.1. Morphology and Microstructure of LSMO Films

**Figure 1:**
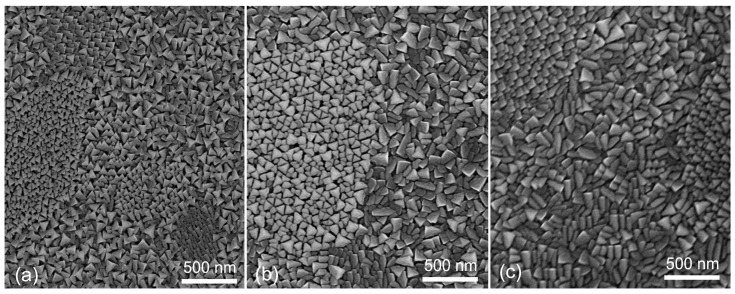
SEM surface images of LSMO films with different Sr content (*x*) and constant content of Mn/(La + Sr) (*y* = 1.15): (**a**) *x* = 0.08, (**b**) *x* = 0.18, (**c**) *x* = 0.3.

SEM images of the surface morphology of the LSMO films grown with different Sr content (*x*) and constant content of Mn excess (*y* = 1.15) are presented in Figure 1. The surface of the films having *x* = 0.08 (see Figure 1a) contain mostly triangular-shaped crystallites with some polygonal-shaped grains. Moreover, some larger islands of different shapes and size of crystallites are observed, which can be caused by the peculiarity of the polycrystalline substrate. It has to be noted that polycrystalline Al_2_O_3_ substrates are made from an ingot that consists of grains of about 2 μm in dimension. When bulk ceramic is cut into plates, the surface of the substrates is formed by grains of different sizes and crystallographic planes. Therefore, during the initial growth process, the LSMO film starts to grow on different planes and sizes of Al_2_O_3_ crystallites, which results in some islands with different shape and size of the crystallites. One can see that some islands show well-ordered crystallites with a triangular shape. As it is known, Al_2_O_3_ has a trigonal Bravais lattice close to hexagonal. Most likely, in these places of the substrate the crystals of Al_2_O_3_ are oriented in the C-plane parallel to the surface, and at the initial stage of the film growth the nuclei are oriented so that their pseudocubic axis [111] is perpendicular to the surface. As a result, a well-oriented film texture is obtained. In other areas of the substrate surface, the crystallites grow with a more disoriented structure. The surface morphology of the films with *x =* 0.18 (Figure 1b) is a little bit different. These films consist of crystallites whose surfaces are not only like triangles, but the elongated rectangular-shaped crystallites start to dominate. A further increase in Sr content (Figure 1c, *x =* 0.30) leads to the dominance of crystallites with this elongated rectangular surface. Moreover, islands with crystallites of a smaller triangular shape also exist in all samples. The average dimensions of the crystallites do not strongly depend on the Sr content. The most frequently found crystallite dimensions are equal to 75 nm for *x* = 0.08, 80 nm for *x* = 1.18, and 85 nm for *x* = 0.3. The change in the shape of the crystallite surface depending on the concentration of Sr atoms can be caused by the deformation of the crystalline lattice or change in the crystalline structure. This can result in the preference of crystallite crystalline orientation in a direction perpendicular to the substrate surface. As a result, a different cut of the crystallite surface takes place.

**Figure 2:**
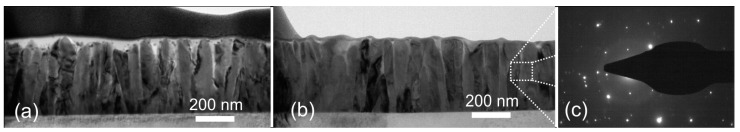
TEM image of the LSMO films with different Sr content (*x*) and constant content of Mn (*y* = 1.15): (**a**) *x* = 0.08, (**b**) *x* = 0.18. (**c**) Selected area electron diffraction pattern from the region shown in (**b**) of film with Sr content *x* = 0.18.

To understand the peculiarities of the growth of the films, TEM images were analyzed. The low magnification cross-sectional TEM images of the films with an Sr content of 0.08 and 0.18 are shown in Figure 2a,b, respectively. As can be seen, the film consists of vertically aligned columns, which are spread throughout the whole film thickness with their long axis arranged perpendicular to the substrate. The typical column width in all films is about 60–75 nm on the upper side and 30–50 nm near the substrate.

For the films with an Sr content of 0.08, the microstructure is columnar, but very porous with both inter- and intracolumnar voids outlining a pronounced dendritic pattern. Moreover, a mixture of columns and separate crystallites are observed in these films, especially in the regions close to the substrate. In addition, the contrast of the TEM images within the columns reveals that the columns are not single crystalline and consist of several single crystal slabs. These slabs produce the macro-steps, which increase the intercolumnar region between crystallites. The increase in the Sr content leads to more perfect structures of the columns and in the grain boundary regions. The lateral surfaces of crystallites and the boundaries between them are flat (see Figure 2b), with no zigzags or steps. Individual crystallites are monocrystalline, although rotated in the plane of the substrate. This is evidenced by the diffraction pattern shown in Figure 2c, in which one can see the spot diffraction pattern from several single crystallites. The films with Sr content *x* = 0.3 are similar in structure to the films with *x* = 0.18.

### 3.2. Resistivity of Nanostructured LSMO Films: Dependence on Sr Content and Excess of Mn

It is known from the literature that the main magnetic and electrical properties of lanthanum manganites could be explained by a double-exchange interaction mechanism between Mn^4+^ and Mn^3+^ ions and a Jahn–Teller effect. One of the characteristics of the films is their resistivity dependence on temperature. These films exhibit a metal–insulator transition typical for manganites at a certain temperature, *T*_m_. For monocrystalline films, this temperature is close to the *Curie* temperature (*T*_C_), and it is believed to be the highest at an Mn^4+^/Mn^3+^ ratio of 1/3 [11]. The doping of manganites with Sr atoms controls this ratio. Moreover, it was demonstrated that even in insulating LaMnO_3_ manganite films, an excess of Mn can induce La vacancies, which result in an increase in Mn^4+^ amount [32]. For nanostructured polycrystalline manganite films, this ratio could be different. It is caused by lattice distortions and defects that are expected during growth, and affect the electrical and magnetic properties of such films. Moreover, for the polycrystalline films, strong variations in the electronic and magnetic properties can be achieved with defects such as La vacancies induced by Mn excess in the films [33].

**Figure 3:**
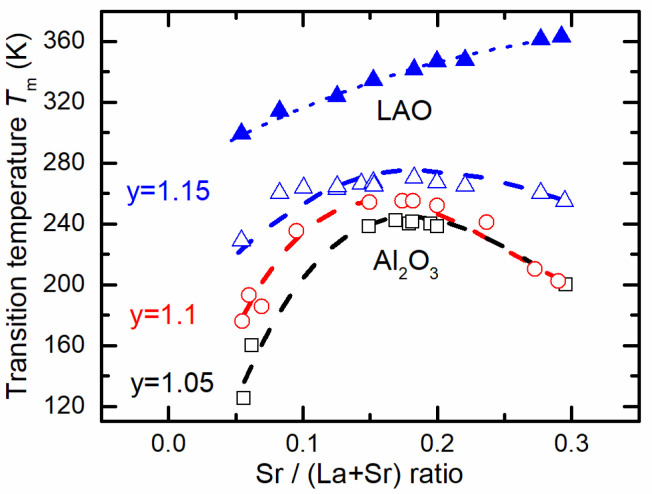
*T*_m_ dependence on Sr content of nanostructured LSMO/Al_2_O_3_ (open symbols) and epitaxial LSMO/LAO (closed symbols) films with different *y* = Mn/(La + Sr) ratio (symbols). Curves—fit to eyes.

Measurements of resistivity vs. temperature dependences of nanostructured LSMO films were performed to investigate the effect of variation in Sr/(La + Sr) and Mn/(La + Sr) ratios on the metal–insulator transition temperature *T*_m_ values. The results are summarized in Figure 3.

There is a tendency that the higher the Mn/(La + Sr) ratio in the films, the higher the *T*_m_ values obtained. Furthermore, at low Sr content, the Mn/(La + Sr) ratio has a stronger influence on the electrical properties. The *T*_m_ versus Sr content has a maximum that slightly shifts to lower Sr values with increasing *y* = Mn/(La + Sr) ratio in the films. It could be explained by induced more La vacancies with the increase in Mn excess, which result in an increase in Mn^4+^ amount [32]; therefore, less Sr is needed for the same Mn^4+^/Mn^3+^ ratio, which is important for transport properties in manganites. The dependence of *T*_m_ on the Mn/(La + Sr) ratio for different Sr content shows that the *T*_m_ values saturate with increasing Mn/(La + Sr) ratio in the films. This indicates that the Mn/(La + Sr) ratio and Sr content *x* values of 1.15 and (0.17–0.18), respectively, result in the highest metal–insulator transition temperatures. It is worth mentioning that for epitaxial films grown on LAO, the maximum *T*_m_ is obtained for an Sr concentration of *x* ≈ 0.3 (see Figure 3). As can be seen, in the range of 0.05 ≤ *x* ≤ 0.3, the *T*_m_ of epitaxial films is continuously increasing. Moreover, it is higher than for films grown on the Al_2_O_3_ substrate. This difference can be caused by the different microstructure of the films. For polycrystalline films, the conducting mechanism is controlled not only by a double-exchange mechanism between manganese ions Mn^3+^–O^2−^–Mn^4+^, but also by the grain boundary resistivity and the relative quantity thereof [34,35]. As a result, the resistivity of the films can vary greatly depending on the disorder of grain boundaries and the relative quantity of their material.

**Figure 4:**
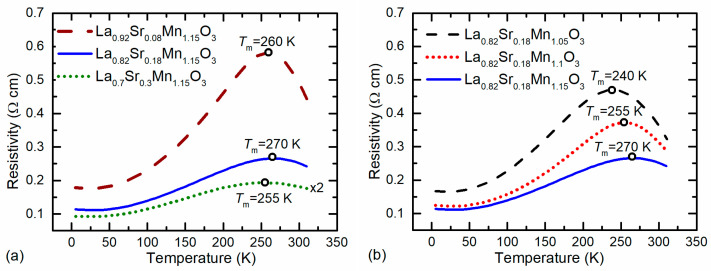
Resistivity vs. temperature dependences of LSMO films having different content of Sr/(La + Sr) (**a**) and Mn/(La + Sr) (**b**).

Figure 4 presents examples of the resistivity *ρ* vs. temperature dependences for LSMO films grown with constant Mn excess while changing Sr content (Figure 4a) and constant Sr content, but different Mn excess (Figure 4b). One can see that all films exhibit a transition from metal-like to an insulator-like resistivity dependence on temperature at a certain critical temperature *T*_m_ corresponding to the resistivity maximum *ρ*_m_ (summary of *T*_m_ values for all investigated films is presented in Figure 3). As it was already mentioned, the increase in Sr content up to *x* = 0.17–0.18, while keeping the Mn content constant, results in an increase in the *T*_m_, while further increase in *x* results in a decrease in *T*_m_. However, the resistivity maximum *ρ*_m_ of these films decreases with the increase in *x* in the whole range of Sr content (0.05 ≤ *x* ≤ 0.3) (see Figure 4a). The resistivity vs. temperature dependences for LSMO films having different Mn excess and a constant *x* = 0.18 are presented in Figure 4b. The *x* = 0.18 was chosen as an average of Sr content at which the films with different Mn excess exhibit maxima of metal–insulator transition temperature (see Figure 3). It can be seen from Figure 4b that for nanostructured LSMO films, *T*_m_ increases while resistivity decreases, with an increase in Mn excess. Our recent study has shown that even higher metal–insulator transition temperature *T*_m_ can be obtained (285 K) with a further increase in Mn content up to 1.21 [36]. However, an increase in Mn *y* > 1.15 causes large nonstoichiometry of the films, and it is difficult to control the homogeneity of the films having only one LSMO phase. For this reason, to ensure a single LSMO phase in the films, we kept the Mn excess in the films not higher than 1.15.

### 3.3. Magnetoresistance of Nanostructured LSMO Films

**Figure 5:**
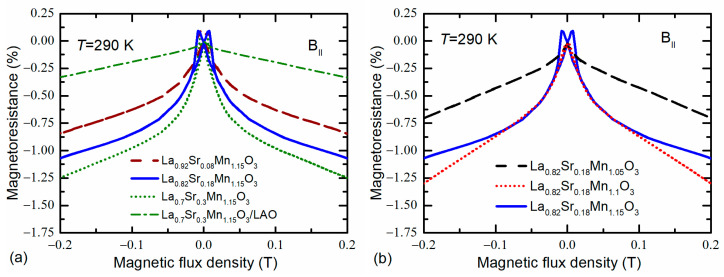
Low-field magnetoresistance dependences on magnetic flux density for nanostructured films with different Sr content (**a**) and Mn excess (**b**). In addition, the dependence for epitaxial LSMO/LAO film (*x* = 0.3, *y* = 1.15) is presented in (**a**) for comparison. Measurements were performed at ambient temperature, 290 K.

The magnetoresistances dependences on magnetic flux density (*B*) measured up to 0.2 T at room temperature (290 K) are presented in Figure 5. The magnetic field was applied in parallel to the film plane. For nanostructured films with Sr content of *x* = 0.18–0.3, a sharp increase in the negative magnetoresistance (the large decrease in electrical resistance) at low fields is observed, which is followed by a slower background negative *MR* with an increase in the magnetic field (see Figure 5a). These effects are usually called the low-field magnetoresistance (*LFMR*) and high-field magnetoresistance (*HFMR*), respectively. Furthermore, one can observe some positive *MR* changes at low magnetic fields, with maxima attributed to the films’ coercive field. In our investigated samples, the *LFMR* was strongly dependent on the concentration of Sr. It can be seen that for films with *x* = 0.3, the *LFMR* is about –0.8% at 50 mT, while for films with *x* = 0.08, the *LFMR* is about –0.48%. At higher fields (0.8 T, not presented in this figure), the *HFMR* was found –4% and –2.5%, respectively. It has to be pointed out that for epitaxial films grown on monocrystalline LAO substrate the *LFMR* was not observed in all investigated films (see Figure 5a for *x* = 0.3). This result could be a confirmation that the origin of the *LFMR* is mostly related to the crystallite–grain boundary structure of the LSMO films, but not with possible rearrangement of the crystal lattice caused by the change in Sr content. For nanostructured films with different Mn excess (Figure 5b), the similar *LFMR* values were obtained for *y* = 1.1 and 1.15, while for 1.05 the *LFMR* was very small. However, at 0.8 T the highest *LFMR* = 4.5% was found for film with Mn excess ratio 1.1. Such increase in *MR* at a higher field could be related with a slightly higher disorder level of grain boundary material (confirmed by higher resistivity values). Therefore, the higher magnetic field could align Mn moments and as a result the resistance change with magnetic field is larger. It should be noted that the *LFMR* is usually not observed in epitaxial films or monocrystalline manganites [20], but found in films with a large number of grain boundaries having a noncollinear spin structure in which spin-polarized transport (tunneling) across the grain boundaries dominates. The *LFMR* is more pronounced at low temperatures and depends on the thickness and type of grain boundaries, because they decouple the neighboring ferromagnetic (FM) grains and provide an energy barrier for spin-polarized tunneling of electrons. The *LFMR* usually vanishes at room temperature, at which the grains become paramagnetic. However, as we can see in Figure 5, this effect can be also obtained at room temperature for the special chemical composition of nanostructured films. Such a result is promising for the application of LSMO films for low magnetic field sensing at room temperature. It is also worth noting that the *LFMR* continuously increases with increase in Sr concentration. This is in contrast to the dependence of *T*_m_ on Sr content for nanostructured films (see Figure 3), for which we observe a decrease in *T*_m_ when *x* > 0.2. Moreover, the Mn excess significantly influences the *LFMR* values of the films. This effect was observed at room temperature only for Mn concentration higher than *y* = 1.1. It can be seen in Figure 5b that for Mn concentration *y* = 1.05 (almost stoichiometric films), the *LFMR* is insignificant.

**Figure 6:**
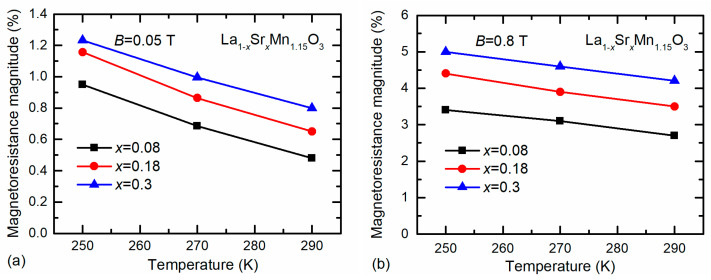
Temperature dependences of magnetoresistance magnitude of nanostructured LSMO films with Mn excess *y* = 1.15 having different Sr content. Measurements were performed at *B* = 0.05 T (**a**) and *B* = 0.8 T (**b**).

As it was already mentioned, the low-field magnetoresistance is more pronounced at low temperatures. The *MR* of nanostructured LSMO films with Mn excess *y* = 1.15 was measured in the temperature range of (250–290) K. Figure 6 presents summarized results obtained at two magnetic flux density values: *B* = 0.05 T (a) and 0.8 T (b). One can see that the *MR* values gradually increase with decrease in temperature and are the highest for the films with *x* = 0.3. Therefore, the nanostructured films with chemical composition La_0.7_Sr_0.3_Mn_1.15_O_3_ could be used for magnetic field sensor applications at room temperature.

To explain the reasons for the observed *LFMR* at room temperature, the studies of the magnetization of the films were carried out.

**Figure 7:**
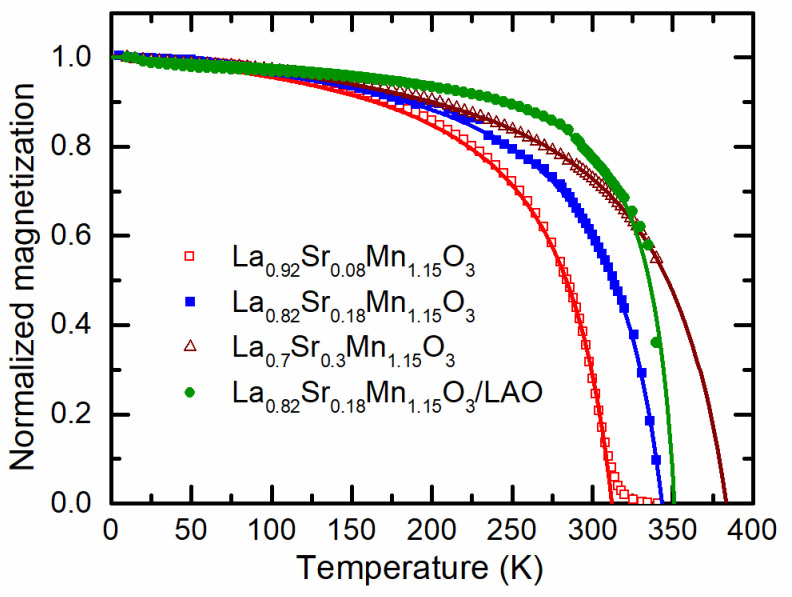
Symbols—temperature dependence of field-cooled (FC) (*H*_FC_ = 1000 Oe) magnetization normalized to magnetization at 10 K for films with different Sr content and constant Mn content *y* = 1.15. Lines—fitting results for the approximation with the *Curie–Weiss* law (1).

Figure 7 presents the temperature dependences of the field-cooled magnetization *M* at a magnetic field of 1000 Oe. For the comparison of different films, the magnetization was normalized to its value at 10 K. One can see that the *M* decreases with increase in temperature, and the ferromagnetic–paramagnetic phase transition is observed in all films. The phase transition temperature (*Curie*, *T*_C_) can be determined from the minimum of derivative d*M*/d*T*. It was obtained that *T*_C_ equals 300 K for the film with Sr concentration of 0.08. In the other cases, due to limited range of the measurement temperatures, we were only able to estimate *T*_C_ from the *Curie–Weiss* law by fitting the experimental data to the formula:

(1) $$M=\frac{a}{T-\theta }+b,$$

where a,b,and θ are fitting constants. The curves in Figure 7 present the fitting results. For the films with *x* = 0.18 and 0.3, the following *T*_C_ were estimated: 343 K and 383 K, respectively. Thus, the *T*_C_ of all films with Mn excess of 1.15 is higher than room temperature, and this explains the *LFMR* effect observed at room temperature. In addition, it should be noted that the *T*_C_ for the film grown on the polycrystalline Al_2_O_3_ and monocrystalline LAO substrates is almost the same, and only the slope of the curves in the region below the transition temperature differs. The studies performed by Chen et al. [37] and Moshnyaga et al. [38] show that the interface strain has a significant effect on the *T*_C_ of the manganite-based composites films. In these studies, the interface strain between the LSMO and NiO nanocolumns causes the LSMO to be in a tensile strain state along the out-of-plane direction, resulting in the change in the length and angle of Mn–O–Mn bonds. The electron transfer integral of the Mn^3+^–O^2−^–Mn^4+^ is decreased and the double-exchange interaction is weakened, leading to the decrease in the *T*_C_. In our case, the films consist of column-shaped crystallites, which are spread throughout the whole film thickness with their long axis arranged perpendicular to the substrate. The typical column width in all films is about 70–80 nm, and the width of the grain boundary is only about 2–3 nm. Such structure with large enough crystallites separated by narrow grain boundaries in the films with higher Mn and Sr content could be the reason why the *T*_C_ of the films grown on monocrystalline and polycrystalline substrates are similar.

**Figure 8:**
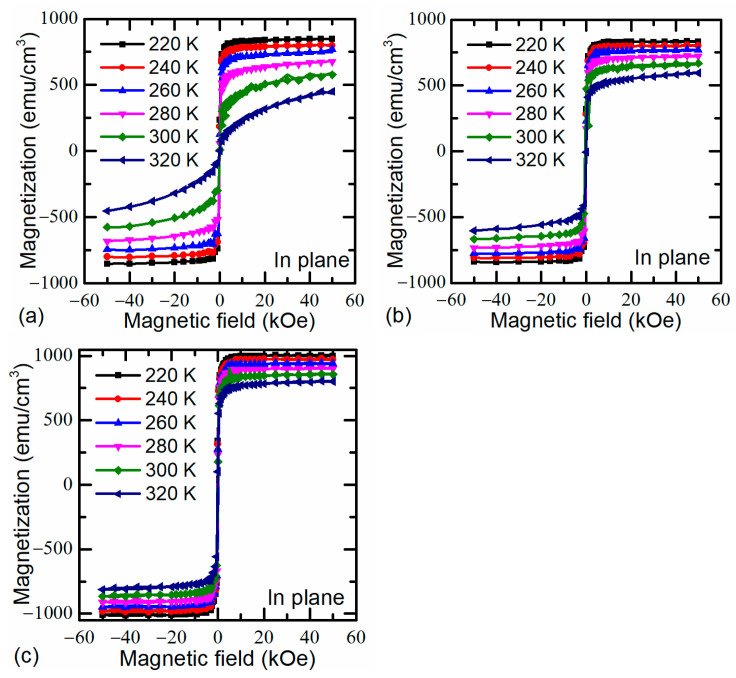
Magnetic hysteresis loops of LSMO films with different Sr content ((**a**) La_0.92_Sr_0.08_Mn_1.15_O_3_, (**b**) La_0.82_Sr_0.18_Mn_1.15_O_3_, (**c**) La_0.70_Sr_0.3_Mn_1.15_O_3_) at temperature range of 320−220K along the in-plane direction.

The magnetic hysteresis loops recorded after field-cooling at 1000 Oe in the temperature range from 320 K down to 220 K of the investigated nonstoichiometric (*y* = 1.15) films are shown in Figure 8. The saturation magnetization (*M_sat_*) of the films with Sr content of 0.08 (Figure 8a) is smaller than that of the LSMO film with *x* = 0.3, which may be attributed to the weakened double-exchange interaction and the spin disorder at crystallite–grain boundary interfaces. For example, at temperature *T* = 220 K the maximal magnetization of the films with *x* = 0.08 equals to *M* = 840 ± 10 emu/cm^3^, when for films with *x* = 0.3 it is 1004 emu/cm^3^. These are quite large values. For comparison, it was shown in [39] that even at a temperature of 10 K the maximal magnetization for stoichiometric La_0.7_Sr_0.3_MnO_3_ manganite films was obtained at only about 550 ± 10 emu/cm^3^. When comparing two films with different Sr content, it can be seen that at 320 K for *x* = 0.08, *M* does not saturate even at the 50 kOe magnetic field, whereas when *x* increases to 0.3, the *M*−*H* loops were saturated already at 10 kOe with *M* = 870 ± 10 emu/cm^3^ at room temperature. It means that at this temperature, films with Sr concentration of *x* = 0.08 are in the paramagnetic phase, while films with *x* = 0.3 and *y* = 1.15 are in the saturated ferromagnetic state. Moreover, the hysteresis of this film at room temperature is very narrow, and a coercive field is approximately 2–3 mT. It should be noted that *LFMR* is associated with a large number of thin grain boundaries having noncollinear spin structure, good magnetic connectivity between the grains, and a high saturation magnetic moment of the grains. To increase the magnitude of *LFMR,* many groups have attempted to grow the manganite-based nanocomposites by incorporating to single-phase films a secondary insulator phase and to introduce the artificial GBs [39,40,41,42,43]. Our research has shown that such results can be achieved without incorporation of the second phase, just by growing single-phase vertically aligned nanostructures with increased excess of Mn and the optimization of the chemical composition Sr/(La + Sr) in the films. The increase in Sr content up to 0.3 when Mn excess in the films exceeds 1.15 leads to an increase in saturation magnetization of the column-like crystallites of the films even at room temperature. Moreover, such composition of the films allows to obtain crystallites with decreased thickness of grain boundaries and improve their quality. As a result, the *LFMR* in such nanostructured films could be observed at room temperature.

For the application of manganite films as low magnetic field sensors, the sensitivity of the measured signal response to magnetic field change is very important. In contrast to GMR sensors, which magnetoresistance saturates at a certain low magnetic field, the magnetoresistance of polycrystalline films exhibiting the CMR effect does not saturate up to very high fields (only the slope of *MR* dependence on *B* is decreased). Therefore, the sensitivity of CMR films can be described as the normalized value of the voltage change across the sensor’s resistance *R*_S_ with an increase in the magnetic field by 1 T: *S* = δ(*V*_out_/*V*_0_)/δ*B*. The maximal value of sensitivity *S* = 137 mV/V·T at room temperature (*T* = 290 K) was obtained almost constant up to 10 mT for the film with *x* = 0.3. Then, it gradually decreased to *S* = 15 mV/V·T at a magnetic flux density *B* > 100 mT. The other films with *x* = 0.18 and *x* = 0.08 showed lower sensitivity values: *S* = 96 mV/V·T and *S* = 85 mV/V·T up 10 mT, respectively. A decrease in the temperature increases the *MR* of the films and their sensitivity as well. The sensitivity of the film with *x* = 0.3 at *T* = 270 K was *S* = 185 mV/V·T up to 10 mT, while at *T* = 250 K *S* = 235 mV/V·T. This provides the opportunity to have more sensitive sensors with external cooling options if higher sensitivity is necessary.

## 4. Conclusions

It was concluded that the content of the doping element Sr and amount of Mn excess in nonstoichiometric nanostructured La_1-x_Sr_x_Mn_y_O_3_ manganite films grown on polycrystalline Al_2_O_3_ substrate has a great influence on the main transport and magnetic properties of these films. It was found that in the range of 0.05 ≤ *x* ≤ 0.3, all films exhibit a metal–insulator transition typical for manganites at a certain temperature, *T*_m_. The maximum value of *T*_m_ is determined by the concentrations of Sr and increases as the ratio *y* = Mn/(La + Sr) increases. It can be caused by the Mn excess, which creates La vacancies that lead to an increase in the Mn^4+^/Mn^3+^ ratio and enhanced ferromagnetic behavior of the films. Moreover, the dependence of *T*_m_ versus Sr content has a maximum at *x =* 0.18 for films with *y* = 1.05, that shifts to lower Sr concentration (*x =* 0.15–0.17) with an increase in Mn excess up to *y =* 1.15. At the same time, the Sr concentration in the films influences the morphology and structure of the crystallites. Films with *x* = 0.3 and *y* = 1.15 have flat lateral surfaces of vertically aligned crystallites and straight boundaries between them without zigzags or steps, which are observed in the case of films with *x* = 0.08. Moreover, contrary to the *T*_m_ change versus Sr content, the highest *Curie* temperature (*T*_C_ = 383 K) is obtained for nanostructured films with chemical composition *x* = 0.3 and *y* = 1.15, as in epitaxial films grown on LAO substrate. The above-mentioned properties have a large effect on the observed increased low-field magnetoresistance of these films. Moreover, the enhanced room temperature *LFMR* could be explained not only by a high *T*_C_ value, but also taking into account the microstructure of the films and high saturation magnetization value (*M* = 870 emu/cm^3^). The *LFMR* effect in these films takes place due to spin-polarized tunneling of charge carriers in high structural quality crystallites, which are in saturated ferromagnetic phase even at room temperature, through thin grain boundaries. As a result, the magnetoresistance of (–1.23 ÷ –0.8)% at temperatures (250 ÷ 290 K) was achieved at a magnetic field of 50 mT for manganite films without the introduction of a secondary insulating phase, and only due to the adjustment of the chemical composition and conditions of film growth.

In summary, it was concluded that vertically aligned nanostructured lanthanum manganite films La_1-x_Sr_x_Mn_y_O_3_ with *x* = 0.3 and Mn excess of *y* = 1.15 grown on polycrystalline Al_2_O_3_ substrates could be used for the development of magnetic field sensors with predetermined parameters for operation at room temperature to measure weak magnetic fields.

## Data Availability

Not applicable.
